# Communication tools and sources of education and information: a national survey of rural and remote nurses

**DOI:** 10.5195/jmla.2019.632

**Published:** 2019-10-01

**Authors:** Julie Kosteniuk, Norma J. Stewart, Erin C. Wilson, Kelly L. Penz, Ruth Martin-Misener, Debra G. Morgan, Chandima Karunanayake, Martha L. P. MacLeod

**Affiliations:** Professional Research Associate, Canadian Centre for Health and Safety in Agriculture, College of Medicine, University of Saskatchewan, Saskatoon, SK, Canada, julie.kosteniuk@usask.ca; Professor Emerita, College of Nursing, University of Saskatchewan, Saskatoon, SK, Canada, norma.stewart@usask.ca; Assistant Professor, School of Nursing, University of Northern British Columbia, Prince George, BC, Canada, erin.wilson@unbc.ca; Assistant Professor, College of Nursing, University of Saskatchewan, Regina Campus, Regina, SK, Canada, kelly.penz@usask.ca; Professor, School of Nursing, Dalhousie University, Halifax, NS, Canada, ruth.martin-misener@dal.ca; Professor, Canadian Centre for Health and Safety in Agriculture, College of Medicine, University of Saskatchewan, Saskatoon, SK, Canada, debra.morgan@usask.ca; Professional Research Associate, Canadian Centre for Health and Safety in Agriculture, College of Medicine, University of Saskatchewan, Saskatoon, SK, Canada, chandima.karunanayake@usask.ca; Professor and Northern Health – UNBC Knowledge Mobilization Research Chair, School of Nursing, University of Northern British Columbia, Prince George, BC, Canada, macleod@unbc.ca

## Abstract

**Objective:**

This study examined accessibility of communication tools in the workplace, use of education to update nursing knowledge, and use of information to make specific decisions in practice among registered nurses (RNs) and licensed practical nurses (LPNs) in rural and remote communities in Canada.

**Methods:**

Data were analyzed from the cross-sectional survey, “Nursing Practice in Rural and Remote Canada II,” of regulated nurses practicing in all provinces and territories of Canada. Data were collected from April 2014 to August 2015.

**Results:**

The survey was completed by 3,822 of 9,622 nurses (40% response), and the present analysis was conducted with a subsample of 2,827 nurses. High-speed Internet was the most accessible communication tool, and nurses used “online/electronic education” more often than “in-person education” to update their nursing knowledge. Internet searches were used more often than several other online/electronic sources to inform decision making. Compared to LPNs, RNs reported greater workplace access to most communication tools and greater use of online/electronic education as well as information sources in online/electronic and print formats. Compared to nurses in community-based health care and hospital settings, nurses in long-term care settings reported lower access to most communication tools, lower use of online/electronic and in-person education, and lower use of online/electronic information.

**Conclusions:**

Access to continuing education and up-to-date information is important for effective patient care. This study points to a need for further research on the continuing education and information needs of rural and remote RNs and LPNs, and on their capacity to incorporate and apply new knowledge in practice.

## INTRODUCTION

Three groups of nursing professionals are regulated to practice in Canada [1]: registered nurses (RNs), including nurse practitioners (NPs); licensed practical nurses (LPNs); and registered psychiatric nurses (RPNs). RNs and LPNs represent 71% and 28% of all regulated nurses in Canada, respectively [1], as well as the majority of regulated nurses in the United States [2]. RNs and LPNs in both countries are required to pass a national exam before licensure; however, licensing and regulatory oversight is provided by states, provinces, and territories [2–6]. Historically, practical nursing programs were created to train LPNs in a short period of time as a way to quickly fill a gap in the RN workforce during World War II [7]. Canadian provinces began to require a 4-year bachelor’s degree for entry to RN practice as recently as 1998, except for Quebec, where a diploma is still the requirement [1, 8]. Forty-five percent of Canadian RNs remain diploma prepared [9]. Educational preparation for RNs in the United States is similar, ranging from a diploma to a 4-year bachelor’s degree [2].

Educational programs for LPNs in Canada, initially one year and now two years, have historically been shorter than those for RNs [10]. Similarly, a one-year program is required for LPN preparation in the United States [2]. Consequently, RNs are expected to have a more developed knowledgebase and clinical skills than LPNs, more advanced critical thinking skills, and a greater capacity for working autonomously and with populations with complex needs [11, 12]. Nevertheless, both RNs and LPNs are expected to build on their knowledgebases after initial training and to maintain their competencies [13].

In Canada, 10% of RNs [14] and 14% of LPNs [15] practice in rural areas, where 17% of the population lives [16]. Compared to LPNs, RNs report broader responsibilities in their rural nursing practice that require more complex and analytical thinking related to several areas [17]. For instance, a greater proportion of rural RNs than LPNs report responsibility for independently making a nursing diagnosis (66% versus 36%), referring patients to other health care practitioners (48% versus 29%), prescribing medication using protocols (30% versus 12%), interpreting laboratory and diagnostic tests (46% versus 25%), supervising or mentoring nursing colleagues (61% versus 32%), and leading a unit or shift (47% versus 31%).

The diversity and challenges in rural communities and work settings compel rural nurses to adapt quickly, think critically, and practice effectively despite restricted resources [18, 19], while applying advanced clinical judgment and skills that allow expanded practice roles [20–22]. Rural nurses must fill multiple roles and provide care to patients with varied acute and chronic conditions from diverse cultures and across all age groups [18, 23], often while practicing without nursing colleagues in the same setting [19]. Although rural nurses practice in varying degrees of professional isolation, there may be opportunities to collaborate with other health care professionals working in the same community or in neighboring communities and to draw support from these professionals [19, 24].

To ensure that rural nurses have the capacity to provide safe and effective care, and to maintain competence, it is important that they have opportunities to update their clinical knowledge and skills through continuing education programs [25]. Rural nurses require education that is relevant to their practice and patient population [19, 25]. Relevant continuing education has been shown to increase self-reported confidence, competence, and job satisfaction among rural nurses and to expand their range of practice [26]. However, studies have noted the limited availability of educational opportunities in rural workplaces [24, 27].

Urban-based learning opportunities pose geographical barriers for rural nurses that require organizational support and resources to be overcome, such as paid time, funding, relief staff, and formal requirements or incentives for continuing education [24, 27–30]. Although distance-based learning with synchronous and asynchronous webinars and virtual classrooms reduces the expense and time of travel [25, 31], organizations must still provide support (e.g., paid time or position coverage and back-up). Moreover, some rural communities in North America have lower access to high-speed Internet than urban centers [32, 33], making distance-based learning challenging [9, 11]. For instance, 52% and 61% of individuals in rural areas of Canada and the United States, respectively, have access to high-speed Internet, defined as a download speed of 25 megabits per second (Mbps) or higher [32, 33]. In comparison, 100% and 96% of those in urban centers of Canada and the United States, respectively, have high-speed Internet access.

Several studies of continuing education and information sources in rural nursing practice have centered exclusively on RNs (e.g., Winters et al. [24], O’Lynn et al. [34], Jukkala at el. [35], and Koessel et al. [36]). When studies included LPNs with other types of nurses in rural practice, most examined nurses as a single group rather than examining LPNs separately, and all of the studies considered questions such as *needs* or *barriers to use* rather than rural nurses’ *use of education or information for practice* (e.g., Fairchild et al. [30], Carter-Templeton and Wu [37], Hodge et al. [38]). Given that the nursing responsibilities and education requirements for LPNs and RNs differ and that LPNs account for over one-quarter of the regulated nursing profession in Canada [1], separate examinations of RN and LPN education and information use are warranted.

Further, many studies of continuing education or information sources in rural nursing practice have focused either on single practice settings (e.g., Jukkala at el. [35], Carter-Templeton and Wu [37], Hodge et al. [38], Mills et al. [39], and Ortiz and Bushy [40]) or have included multiple settings but have not analyzed outcomes by setting (e.g., Winters et al. [24], Olade [29], O’Lynn et al. [34], Koschel et al. [41]). When studies have compared outcomes across rural practice settings, the outcomes have included learning needs or attitudes to evidence-based practice rather than nurses’ use of education or information (e.g., Fairchild et al. [30] and Koessel et al. [36]).

Given how little is known about variations in education and information use by practice setting and type of rural nurse, the purpose of the present analysis was threefold. First, the authors investigated the accessibility of communication tools in the primary workplaces of rural and remote RNs and LPNs in Canada. Second, we examined the frequency with which rural and remote RNs and LPNs used education sources to update nursing knowledge and information sources to make specific decisions in nursing practice. Third, we analyzed the findings by registration status (RNs versus LPNs) and practice setting (community-based health care, hospital, and long-term care).

## METHODS

### Design

This project used data from the cross-sectional “Nursing Practice in Rural and Remote Canada II (RRNII)” survey of regulated nurses residing in rural and remote communities across all ten provinces and three territories of Canada. The RRNII survey replicated and extended the first “Nature of Nursing Practice in Rural and Remote Canada (RRNI)” cross-sectional survey [42]. MacLeod et al. provide further details about the RRNII study design and methods [43].

### Study population and sample

For the RRNII survey, work postal codes or home postal codes, when work codes were unavailable, were used to systematically sample rural RNs, LPNs, and RPNs in all 10 Canadian provinces. All rural and remote NPs were also included in the sample. Communities across the 10 Canadian provinces with a core population of less than 10,000 people and a commuting segment of less than 50% of the employed core, were considered rural [44]. All regulated nurses in the 3 territories were considered remote and were included in the sample.

### Data collection

Using the Dillman method of persistent follow-up [45], data were collected by paper and online surveys in English and French from April 2014 to August 2015. A total of 10,072 nurses were sampled; 9,622 were eligible for the survey (i.e., practiced in a rural or remote community at the time of the survey and on leave for 6 months or less) and 450 were ineligible (e.g., worked in an urban area, address incorrect). A total 3,822 of 9,622 nurses completed a survey (response rate of 40%), including 2,082 RNs, 1,370 LPNs, 163 NPs, and 207 RPNs.

The present analysis included 2,827 nurses: 1,646 RNs (excluded n=436) and 1,181 LPNs (excluded n=189). Nurses were included if they were employed in nursing and excluded if they were on leave or retired; held a primary position as an educator, researcher, consultant, or analyst; or were primarily employed in an educational institution, professional association or government, or workplace other than those described below. Also excluded from this analysis were NPs and RPNs, due to the small samples.

### Measures

#### Outcome measures

Informed by previous studies [46–48], this study assessed several main outcomes (supplemental appendix). One outcome measure, “direct access to communication tools in the primary workplace for use in nursing practice,” was assessed using 5 items (yes/no). All other outcomes were assessed with questions regarding how often nurses used specific sources of education and information. Higher scores indicated more frequent use, with all items scored on a 6-point scale from 1 (never), 2 (less than once a year), 3 (at least once a year), 4 (at least once a month), 5 (at least once a week), to 6 (daily). Outcomes measures of “online/electronic education use to update nursing knowledge” and “in-person education use to update nursing knowledge” were each assessed using 1 item. As part of this item, examples of education sources were provided to respondents and included in-service training, workplace education, continuing education, journal clubs, or nursing associations or colleges.

The outcome “online/electronic information use to make specific decisions in nursing practice” was measured with 7 items. A summated score was created by summing the scores for all 7 items, with total scores ranging from 7 to 42 and higher scores indicating more frequent use of online/electronic information. The Cronbach’s alpha estimate for all 7 items was 0.84. The outcome “print/paper information use to make specific decisions in nursing practice” consisted of 4 items. The scores of all 4 items were summed to create a summated score; more frequent use of print/paper information was reflected by higher scores. The Cronbach’s alpha estimate for all 4 items was 0.78. Lastly, frequent users of education or information were defined as nurses who used an education or information source daily or at least once a week and infrequent users were those who used a source at least once a month, at least once a year, less than once a year, or never.

#### Independent variables

The 8 independent demographic and professional practice variables consisted of sex (female or male); age; registration status (RN or LPN); highest nursing education credential (diploma, bachelor’s, master’s, or doctorate); primary position (staff nurse, nurse practitioner, clinical nurse specialist, or manager); living and working in same community (yes or no); and population of primary work community (under 1,000, 1,000–9,999, and 10,000 or over). Practice setting included 13 choices recoded to a single variable with 3 categories (community-based health care, hospital, and long-term care).

Nine practice issue measures were also included as independent variables. The scope of practice variable asked respondents to indicate whether they thought their roles were below, within, or above their registered or licensed scope of practice. The 4-point work confidence item asked respondents to describe their levels of confidence in their work from 1 (extremely low) to 4 (extremely high). Scores on the single-item satisfaction with current nursing practice measure ranged from 1 (strongly disagree) to 5 (strongly agree), with higher scores indicating greater satisfaction.

Nine items were included from the Utrecht Work Engagement Scale [49], each scored from 0 (never/a few times a year) to 6 (always/everyday). Total scale scores ranged from 0 to 54, and higher scores indicated greater work engagement. The original Organizational Commitment Scale [50] was adapted and reduced from 18 to 12 items, each scored from 1 (strongly disagree) to 7 (strongly agree). After reverse scoring 3 items, total scale scores ranged from 12 to 84 and higher scores indicated greater workplace commitment.

Three subscales of the Job Resources in Nursing (JRIN) Scale and 1 subscale of the Job Demands in Nursing Scale (JDIN) were included in this analysis [51]. The 3 JRIN subscales (staffing and time; technology; and training, professional development, and continuing education) and 1 JDIN subscale (preparedness/scope of practice) each consisted of 4 items that were scored from 1 (strongly disagree) to 5 (strongly agree), with subscale scores ranging from 4 to 20 and higher scores indicating a higher perceived level of the resource or demand (some JDIN-preparedness items required reverse scoring).

### Statistical analyses

Statistical analysis was conducted with SPSS version 24.0. Descriptive statistics (frequency, mean, and standard deviation [SD]) were calculated by registration status (RNs and LPNs) and practice setting (community-based health care, hospital, and long-term care). Significant differences (*p*<0.05) by registration status were identified with the χ^2^ test for nominal variables. Pairwise comparisons by practice setting were also examined with the χ^2^ test. We used 2-way analysis of variance (ANOVA) with 2 factors—registration status and practice setting—to examine main effects and interaction for 4 separate interval variables: online/electronic education to update nursing knowledge, in-person education to update nursing knowledge, summated online/electronic information to make specific decisions, and summated print information to make specific decisions. The Tukey post hoc test was used to examine pairwise differences across the 3 practice setting groups, for each of the 4 outcomes.

We selected the source used most often by nurses to update knowledge (online/electronic education) and the source used most often to make specific decisions in nursing practice (Internet search engines) to examine differences in practice issues according to frequency of use. Practice issues were compared for significant differences (*p*<0.05) with the χ^2^ test for nominal variables and Student’s *t*-test for interval variables across 2 groups (frequent and infrequent users) for RNs and LPNs separately.

### Ethics approval

Approval was received by the university ethics boards of research team members in the provinces (British Columbia, Alberta, Saskatchewan, Ontario, Quebec, and Nova Scotia) and research licensing boards in the Northwest Territories and Nunavut.

## RESULTS

### Sample characteristics

Respondents were, on average, 46.6 years of age (SD=11.5, range=20–84). RNs were 47.0 years of age on average (SD=11.6, range=22–84), and LPNs were 46.1 years (SD=11.3, range=20–70). The majority of respondents were female, worked in the same community where they lived, and worked in communities with populations less than 10,000 (Table 1). A similar proportion of RNs held a bachelor’s degree as their highest credential (48.4%) as held a diploma (49.4%), and nearly all LPNs held a diploma as their highest credential (99.6%). Most RNs and LPNs were staff nurses, and the majority were employed in hospitals.

**Table 1:** Characteristics of respondents

Characteristics	Total		Registered nurse (RN)		Licensed practical nurse (LPN)	

	n	(%)	n	(%)	n	(%)
Registration status						
RN	1,646	(58.2%)	1,646	(100.0%)	0	(—)
LPN	1,181	(41.8%)	0	(—)	1,181	(100.0%)
Total	2,827		1,646		1,181	
Sex						
Male	166	(6.0%)	99	(6.1%)	67	(5.9%)
Female	2,592	(94.0%)	1,515	(93.9%)	1,077	(94.1%)
Total	2,758		1,614		1,144	
Highest attained nursing education						
Diploma	1,973	(70.5%)	802	(49.4%)	1,171	(99.6%)
Bachelor’s	790	(28.2%)	785	(48.4%)	5	(0.4%)
Master’s	34	(1.2%)	34	(2.1%)	0	(—)
Doctorate	1	(<0.1%)	1	(<0.1%)	0	(—)
Total	2,798		1,622		1,176	
Primary position						
Staff nurse	2,464	(87.2%)	1,334	(81.0%)	1,130	(95.7%)
Nurse practitioner	116	(4.1%)	101	(6.1%)	15	(1.3%)
Manager	247	(8.7%)	211	(12.8%)	36	(3.0%)
Total	2,827		1,646		1,181	
Practice setting						
Community-based health care	746	(26.4%)	595	(36.1%)	151	(12.8%)
Hospital	1,454	(51.4%)	863	(52.4%)	591	(50.0%)
Long-term care	627	(22.2%)	188	(11.4%)	439	(37.2%)
Total	2,827		1,646		1,181	
Live and work in same community	1,578	(56.9%)	966	(59.7%)	612	(53.0%)
Total	2,772		1,618		1,155	
Population of primary work community						
Under 1,000	368	(13.5%)	230	(14.4%)	138	(12.2%)
1,000–9,999	1,523	(55.8%)	861	(54.0%)	662	(58.4%)
10,000 and over	836	(30.7%)	503	(31.6%)	333	(29.4%)
Total	2,727		1,594		1,133	

Note: Sample sizes vary due to missing values.

### Direct access to communication tools in workplace

Overall, the most accessible workplace communication tool was high-speed Internet (86.3%) and the least accessible was web conferencing such as Skype or WebEx (30.8%) (Table 2). Most nurses reported that they had access to electronic communication among providers such as email or text (80.2%), as well as teleconference (70.2%) and videoconference tools (54.4%). Access varied by nurse group, with direct workplace access to most communication tools reported by a greater proportion of RNs than LPNs. Group differences in access to high-speed Internet were not reported.

**Table 2:** Direct access to communication tools, by registration status and practice setting

Communication tool	Registration status								Practice setting							

	Total		RN		LPN		Chi-square	*p* value	Community-based health care		Hospital		Long-term care		Chi-square	*p* value
	
	n	(%)	n	(%)	n	(%)			n	(%)	n	(%)	n	(%)		
High-speed Internet	2,439	(86.3%)	1,436	(87.2%)	1,003	(84.9%)	3.11	0.078	627	(84.0%)	1,293	(88.9%)	519	(82.8%)	18.25	**<0.001**
Electronic communication among providers	2,266	(80.2%)	1,391	(84.5%)	875	(74.1%)	46.92	**<0.001**	657	(88.1%)	1,176	(80.9%)	433	(69.1%)	78.39	**<0.001**
Teleconference	1,985	(70.2%)	1,267	(77.0%)	718	(60.8%)	86.06	**<0.001**	573	(76.8%)	1,072	(73.7%)	340	(54.2%)	100.73	**<0.001**
Videoconference	1,537	(54.4%)	1,009	(61.3%)	528	(44.7%)	76.31	**<0.001**	448	(60.1%)	868	(59.7%)	221	(35.2%)	118.76	**<0.001**
Web conferencing	870	(30.8%)	589	(35.8%)	281	(23.8%)	46.4	**<0.001**	304	(40.8%)	407	(28.0%)	159	(25.4%)	48.77	**<0.001**

Note: Total (n=2,827), RN (n=1,646), and LPN (n=1,181); community-based health care (n=746), hospital (n=1,454), and long-term care (n=627). *p* values <0.05 are bolded.

Direct workplace access to communication tools also varied by practice setting (Tables 2 and 3). Access to high-speed Internet was greater in hospital settings than community-based health care and long-term care settings. Nurses in community-based health care settings had greater access to electronic communication among providers and to web conferencing than those in other settings. Moreover, access to electronic communication between providers was higher in hospitals than long-term care settings. Teleconference and videoconference access was lower in long-term care settings compared to other settings.

**Table 3:** Direct access to communication tools, by practice setting

Communication tool	Community-based health care vs. hospital		Community-based health care vs. long-term care		Hospital vs. long-term care	

	Chi-square	*p* value	Chi-square	*p* value	Chi-square	*p* value
High-speed Internet	10.57	**0.001**	0.4	0.527	14.73	**<0.001**
Electronic communication among providers	18.34	**<0.001**	75.24	**<0.001**	34.91	**<0.001**
Teleconference	2.48	0.115	77.99	**<0.001**	76.3	**<0.001**
Videoconference	0.026	0.872	83.91	**<0.001**	104.99	**<0.001**
Web conferencing	36.69	**<0.001**	36.11	**<0.001**	1.53	0.216

Note: Total (n=2,827), community-based health care (n=746), hospital (n=1,454), and long-term care (n=627). *p* values <0.05 are bolded.

### Use of education sources to update nursing knowledge

Nurses overall used online/electronic education more often than in-person education to update nursing knowledge (Table 4). Two-way ANOVA analyses showed that registration status had a main effect on the use of online/electronic education to update nursing knowledge (Table 5), with use higher by RNs than LPNs. Practice setting had main effects on the use of both online/electronic and in-person education to update nursing knowledge. Post hoc tests showed more frequent use of online/electronic education in community-based health care compared to hospital and long-term care settings, as well as greater use in hospital compared to long-term care. Furthermore, in-person education was used more often in community-based health care and hospital settings than in long-term care settings. There were no significant interaction effects on the use of either education source.

**Table 4:** Direct access to communication tools, by practice setting

Education or information source	Total		Registration status				Practice setting					

			RN		LPN		Community-based health care		Hospital		Long-term care	

	M	(SD)	M	(SD)	M	(SD)	M	(SD)	M	(SD)	M	(SD)
Education to update nursing knowledge												
Online/electronic	4.0	(1.2)	4.2	(1.1)	3.7	(1.3)	4.3	(1.1)	4.0	(1.2)	3.6	(1.3)
In-person	3.6	(1.1)	3.7	(1.1)	3.5	(1.1)	3.7	(1.2)	3.6	(1.0)	3.5	(1.1)
Online/electronic information to make specific decisions in nursing practice												
Internet search engines (e.g., Google)	4.8	(1.3)	4.9	(1.2)	4.6	(1.4)	5.0	(1.1)	4.8	(1.2)	4.3	(1.5)
Policies, protocols, standards, or regulatory tools	4.4	(1.5)	4.7	(1.3)	4.1	(1.5)	4.7	(1.3)	4.5	(1.4)	3.9	(1.6)
Clinical practice guidelines	4.1	(1.5)	4.4	(1.4)	3.8	(1.5)	4.5	(1.4)	4.2	(1.4)	3.7	(1.6)
Nursing/medical textbooks	3.4	(1.5)	3.5	(1.4)	3.4	(1.5)	3.6	(1.5)	3.4	(1.4)	3.3	(1.5)
Nursing/medical journals	3.3	(1.4)	3.5	(1.3)	3.1	(1.5)	3.7	(1.3)	3.2	(1.4)	3.1	(1.5)
Practice support resources (e.g., NurseOne)	3.2	(1.8)	3.4	(1.8)	2.8	(1.8)	3.6	(1.8)	3.2	(1.8)	2.7	(1.6)
Research databases (e.g., CINAHL)	2.7	(1.6)	2.9	(1.6)	2.5	(1.7)	3.1	(1.6)	2.7	(1.6)	2.4	(1.6)
Summated online/electronic information use	26.0	(7.5)	27.1	(6.9)	24.3	(7.9)	27.6	(7.0)	25.5	(7.3)	22.9	(8.1)
Print information to make specific decisions in nursing practice												
Policies, protocols, standards, or regulatory tools	4.3	(1.4)	4.4	(1.4)	4.1	(1.4)	4.4	(1.4)	4.3	(1.5)	4.2	(1.4)
Clinical practice guidelines	4.0	(1.5)	4.1	(1.5)	3.8	(1.5)	4.2	(1.5)	3.9	(1.5)	3.9	(1.5)
Nursing/medical textbooks	3.5	(1.4)	3.5	(1.4)	3.5	(1.4)	3.7	(1.4)	3.3	(1.4)	3.6	(1.3)
Nursing or medical journals	3.1	(1.4)	3.2	(1.4)	3.0	(1.4)	3.3	(1.4)	3.0	(1.4)	3.2	(1.4)
Summated print information use	14.8	(4.5)	15.2	(4.4)	14.3	(4.6)	15.3	(4.5)	14.3	(4.5)	14.7	(4.5)

Note: M=mean; SD=standard deviation. Scoring for single items: 1=never, 2=less than once a year, 3=at least once a year, 4=at least once a month, 5=at least once a week, 6=daily. Samples vary due to missing cases: total (n=2,505–2,723), RN (n=1,489–1,592), LPN (n=1,016 to 1,132); community-based health care (n=661–720), hospital (n=1,290–1,403), and long-term care (n=554–605). *p* values <0.05 are bolded.

**Table 5:** Use of education and information, two-way analysis of variance (ANOVA) results for registration status and practice setting main effects and interaction

	Online/electronic education to update nursing knowledge				In-person education to update nursing knowledge				Summated online/electronic information use to make specific decisions				Summated print information use to make specific decisions			

	*df*	*F*	*p*	*η2*	*df*	*F*	*p*	*η2*	*df*	*F*	*p*	*η2*	*df*	*F*	*p*	*η2*
a. ANOVA																
Between subjects																
Registration status	1	62.77	**<0.001**	0.023	1	3.31	0.069	0.001	1	30.83	**<0.001**	0.012	1	22.76	**<0.001**	0.009
Practice setting	2	17.57	**<0.001**	0.013	2	3.61	**0.027**	0.003	2	29.08	**<0.001**	0.023	2	6.31	**0.002**	0.005
Registration status × Practice setting	2	1.79	0.167	0.001	2	1.17	0.312	0.001	2	0.301	0.74	<0.001	2	0.73	0.482	0.001
Within-group																
Mean square	2,706	−1.39			2,717	−1.17			2,499	−52.37			2,735	−20.17		
b. Pairwise comparisons																
Community-based primary health care vs. hospital			**<0.001**					0.726			**<0.001**				**<0.001**	
Community-based primary health care vs. long-term care			**<0.001**					**0.003**			**<0.001**				**0.049**	
Hospital vs. long-term care			**<0.001**					**0.007**			**<0.001**				0.125	

Note: Results analyzed by two-way ANOVA followed by Tukey test. Values enclosed in parentheses represent mean square errors. *p* values <0.05 are bolded.

### Use of information sources to make specific decisions in nursing practice

As shown in Table 4, the rank ordered frequency of online/electronic information use for the purpose of making specific decisions in practice was similar between RNs and LPNs. In descending order, the rankings were: Internet search engines (e.g., Google, Yahoo); policies, protocols, standards, or regulatory tools (hereafter referred to as policies or protocols); clinical practice guidelines; nursing or medical textbooks; nursing or medical journals; practice support resources (e.g., NurseOne, UpToDate, eMedicine); and research databases (e.g., CINAHL, Medline, PubMed). The rank ordered frequency of print use was also similar between both nurse groups, following a descending order: policies or protocols, clinical practice guidelines, nursing or medical textbooks, and nursing or medical journals.

Two-way ANOVA analyses indicated that registration status had a main effect on the use of both online/electronic information and print information to make specific decisions (Table 5), with summated scores for both outcomes higher among RNs than LPNs. Practice setting also had a main effect on both outcomes, with Tukey post hoc tests showing a higher summated score on online/electronic information in community-based health care compared to other settings and in hospitals compared to long-term care workplaces (Table 5). Likewise, the summated score for print information use was higher in community-based health care compared to other settings. There were no significant interaction effects on the total use of all sources, regardless of format.

### Practice issues

To determine whether practice issues varied by the frequency with which certain sources were used, we identified the education source used most often to update nursing knowledge (online/electronic) and the information source used most often to make specific decisions in nursing practice (Internet search engines). Among RNs, frequent users of online/electronic education to update knowledge and frequent users of Internet searches to make specific decisions in practice demonstrated greater work engagement than infrequent users (Table 6). Further, frequent users of Internet searches to make decisions in practice indicated higher levels of technology as a practice resource than infrequent users.

**Table 6:** Practice issues among registered nurses (RNs) by frequency of online/electronic education and Internet search engine use

Practice issue	Online/electronic education to update nursing knowledge					Internet search engines to make specific decisions in nursing practice				

	Frequent use		Infrequent use		*p* value	Frequent use		Infrequent use		*p* value
	n	(%)	n	(%)		n	(%)	n	(%)	
	
Current role is within or above registered or licensed scope of practice	580	(94.3%)	915	(94.6%)	0.821	1,066	(94.4%)	423	(94.65)	1
Work confidence (somewhat or extremely high)	587	(95.4%)	923	(95.7%)	0.801	1,078	(95.7%)	426	(95.5%)	0.892
	**M**	**SD (range)**	**M**	**SD (range)**		**M**	**SD (range)**	**M**	**SD (range)**	
	
Satisfaction with current nursing practice	4.0±0.8	(1–5)	4.0±0.8	(1–5)	0.178	4.0±0.8	(1–5)	3.9±0.8	(1–5)	0.156
Work engagement	40.0±9.0	(9–54)	37.6±9.4	(0–54)	**<0.001**	38.9±9.1	(2–54)	37.5±9.8	(0–54)	**0.006**
Organizational commitment	51.1±11.6	(12–84)	51.6±10.6	(14–84)	0.385	51.1±11.1	(12–84)	52.1±10.6	(14–84)	0.091
Practice resource: staffing and time	11.8±3.7	(4–20)	11.7±3.6	(4–20)	0.68	11.8±3.6	(4–20)	11.8±3.6	(4–20)	0.901
Practice resource: technology	13.4±3.5	(4–20)	13.2±3.2	(4–20)	0.18	13.4±3.3	(4–20)	13.0±3.4	(4–20)	**0.047**
Practice resource: training, professional development, and continuing education	12.8±3.6	(4–20)	12.5±3.5	(4–20)	0.13	12.6±3.6	(4–20)	12.6±3.4	(4–20)	0.863
Practice demand: preparedness or scope of practice	7.8±2.1	(4–17)	7.8±1.9	(4–16)	0.604	7.8±2.1	(4–17)	7.9±1.8	(4–16)	0.356

Note: *p* values calculated by χ^2^ test for nominal variables and Student’s *t*-test for interval variables. *p* values <0.05 are bolded. Samples vary due to missing cases: Online/electronic education frequent use (n=577–615), infrequent use (n=895–968); Internet search engines frequent use (n=1,063–1,131), infrequent use (n=404–448).

Among LPNs, frequent users of online/electronic education to update knowledge indicated higher levels of practice resources related to technology and training or professional development than infrequent users (Table 7). Compared to infrequent users of Internet searches to make decisions in practice, frequent users reported higher levels of work engagement and technology as a practice resource.

**Table 7:** Practice issues among licensed practical nurses (LPNs) by frequency of online/electronic education and Internet search engine use

Practice issue	Online/electronic education to update nursing knowledge					Internet search engines to make specific decisions in nursing practice				

	Frequent use		Infrequent use		*p* value	Frequent use		Infrequent use		*p* value
	n	(%)	n	(%)		n	(%)	n	(%)	
	
Scope of practice (current role is within or above registered or licensed scope of practice	225	(83.0%)	693	(82.0%)	0.784	584	(82.0%)	329	(81.6%)	0.872
Work confidence (somewhat or extremely high)	253	(94.1%)	784	(93.0%)	0.675	663	(93.5%)	370	(92.0%)	0.393
	**M**	**SD (range)**	**M**	**SD (range)**		**M**	**SD (range)**	**M**	**SD (range)**	
	
Satisfaction with current nursing practice	3.8±0.9	(1–5)	3.8±0.8	(1–5)	0.825	3.8±0.9	(1–5)	3.9±0.8	(1–5)	0.164
Work engagement	38.7±9.3	(5–54)	37.8±9.6	(0–54)	0.164	38.5±9.4	(0–54)	37.1±9.7	(7–54)	**0.017**
Organizational commitment	53.6±11.0	(27–81)	52.9±10.4	(18–84)	0.34	53.2±10.7	(18–81)	52.9±10.3	(24–84)	0.639
Practice resource: staffing and time	11.5±3.8	(4–20)	11.0±3.4	(4–20)	0.068	11.1±3.6	(4–20)	11.1±3.4	(4–20)	0.864
Practice resource: technology	13.7±3.2	(4–20)	13.1±3.0	(4–20)	**0.014**	13.5±3.0	(4–20)	12.8±3.1	(4–20)	**0.001**
Practice resource: training, professional development, and continuing education	13.1±3.3	(4–20)	12.4±3.4	(4–20)	**0.003**	12.7±3.4	(4–20)	12.4±3.3	(4–20)	0.126
Practice demand: preparedness or scope of practice	7.6±2.0	(4–15)	7.8±2.0	(4–20)	0.057	7.7±2.1	(4–20)	7.9±1.7	(4–14)	0.151

Note: *p* values calculated by χ^2^ test for nominal variables and Student’s *t*-test for interval variables. *p* values <0.05 are bolded. Samples vary due to missing cases: Online/electronic education frequent use (n=249–270) and infrequent use (n=753–841); Internet search engines frequent use (n=660–709) and infrequent use (n=340–401).

## DISCUSSION

Our study found a number of patterns in access to communication tools and use of education and information among rural and remote nurses. First, direct workplace access to five communication tools followed similar patterns for RNs and LPNs, with high-speed Internet the most accessible and web conferencing the least accessible tools. Additionally, both nurse groups more often drew on online/electronic than in-person education sources to update their nursing knowledge. Finally, the pattern of information use for decision making in nursing practice was similar across both nurse groups: Internet searches were most popular and research databases least popular in terms of online/electronic information, and policies or protocols were most popular and nursing or medical journals least popular in terms of print information.

Our finding that high-speed Internet was accessible to 86% of rural and remote nurses overall corresponds to previous studies that found a considerable majority of rural RNs have workplace Internet access [24, 34]. However, it is worth noting that 1 in 10 nurses in our study lacked workplace access to high-speed Internet. Further, web conferencing, an important tool for synchronous online learning [52], was directly accessible in a small minority of workplaces (31% overall). Web conferencing tools were not defined for respondents in our study; however, the Skype and WebEx applications were provided as examples. Although taking part in web conferences generally requires little more than Internet access in terms of technology, it is also essential for participation that users have adequate time, adequate workspace, and a reliable Internet connection. Communication technology can reduce the professional and social isolation of rural nurses [31], address the challenges of travel to access urban-based in-person education [53], and support skill development through online learning [25], but these tools must first be accessible to nurses in their workplaces.

The higher use of online/electronic education than in-person education among rural and remote nurses in the present analysis suggests that online/electronic education formats are more accessible and/or relevant than face-to-face formats. A recent US study of rural hospital nurses, mainly RNs, found that nurses preferred in-person education, with online courses second in choice over other methods such as self-instruction and videoconferencing [38]. However, an early US study of mainly hospital-based rural RNs found that budget cuts meant fewer in-service opportunities, and external face-to-face education required travel to a city [24]. While in-person formats may be preferred, web-based formats alone or in conjunction with a face-to-face component [26] are perhaps more accessible than face-to-face alone, require less travel, may be more convenient and cost-efficient, and offer a range of educational opportunities. Slow Internet connections and lack of topic relevance, in addition to heavy workloads in understaffed workplaces, are barriers that remain to be addressed when offering online continuing education to rural nurses [26, 28].

The online/electronic and print information sources that rural and remote RNs and LPNs in this study reported using most and least often warrant closer examination. Considering online/electronic sources, Internet search engines were used most often, followed by policies or protocols, clinical practice guidelines, textbooks, journals, practice support resources, and research databases. The use of print sources followed a similar pattern, with nurses reporting most frequent use of policies or protocols, followed by clinical practice guidelines, textbooks, and journals.

A report from the larger RRNII study indicates that following protocols or decision support tools to arrive at a plan of care is among the top four practice responsibilities that rural and remote RNs and LPNs reported (ranked by frequency) [17]. This suggests that the use of policies or protocols and clinical practice guidelines is an integral part of practice for both nurse groups. The only information source used more often than online policies or protocols by both nurse groups, according to the present study, was Internet searching.

The top ranking of Internet search engines was similarly found in a study of newly graduated LPNs in one Canadian province [54]. Where rural nurses work alone or with few colleagues [55, 56], an Internet search may be an accessible alternative to consulting a colleague. An Internet search can offer information that is relevant to rural practice in a convenient and more timely manner than a search of online/electronic textbooks, journals, and databases. Furthermore, research databases and academic journals that require subscriptions might not be widely available to many rural nurses. Nevertheless, the low use of online/electronic practice support resources and research databases in our results, relative to higher use of Internet search engines such as Google, suggests that rural and remote nurses are accessing non-peer-reviewed information of questionable quality to a greater extent than research-based information. Evidence suggests that the main barriers to using research literature (e.g., electronic database subscriptions) among nurses include time, skill, and access [57].

While RN and LPN similarities were apparent in our research, distinct differences also emerged. First, RNs were more likely than LPNs to report direct workplace access to all communication tools, except for high-speed Internet, which was equally accessible to both groups. Second, RNs more often than LPNs drew on online education to update their nursing knowledge, as well as online and print information to make decisions in nursing practice (except for textbooks). Among LPNs who were infrequent users compared to frequent users of online/electronic education to update their nursing knowledge, we found perceptions of lower use of resources related to training/professional development and technology (e.g., access to electronic resources).

These findings correspond to evidence suggesting that some new LPN graduates, the majority of whom hold two-year diplomas, have not been well prepared to identify, access, and evaluate the most appropriate research for practice [54]. The expectation that LPNs will use research evidence to the same extent as RNs in nursing practice varies across Canadian LPN regulatory groups [10]. Our findings point to a need for future research on the continuing education and information needs of LPNs and RNs relative to their positions and workplaces, particularly in rural and remote communities where nurses may work alone or with few colleagues to consult. Given their different educational requirements and practice responsibilities, further examination of LPN and RN preparation and capacity to apply new knowledge in practice is also warranted.

For nurses in this study, direct access to electronic communication among providers and teleconference and videoconference tools were lowest in long-term care practice settings. Long-term care practice settings also demonstrated the lowest use of online/electronic and in-person education to update nursing knowledge and the lowest use of online/electronic information to make specific decisions in nursing practice. A study of rural and urban long-term care facilities in a Canadian province found that access and incentives to take part in continuing education were important enablers, but the success of continuing education programs was largely determined by organizational support such as adequate resources and opportunities to subsequently implement new care initiatives [56]. Stolee et al. emphasize that although training is necessary, it is not sufficient to improve patient care [58]. Staff must feel empowered to change practice, and the workplace must share a common goal of quality improvement. Further research to investigate variations in the use and uptake of education and information across rural health care practice settings is merited.

The results of our research suggest that work engagement and technology availability in the workplace are important correlates of education and information use among rural and remote nurses. It is possible that information technology availability and workplace engagement are associated; however, we did not examine this relationship in the present analysis. Others have noted the important role of rural workplaces in facilitating research use by providing technology, including computers and Internet access, as well as time to search for information and take part in continuing education programs [24, 25, 34]. Williams further suggests that information technology may reduce professional isolation and, in turn, improve retention of rural nurses [31].

### Limitations

This analysis has some limitations that should be noted. Our sample was representative of rural Canadian RNs and LPNs [17, 59]; however, our findings might not be generalizable to rural nurses outside of Canada. The survey was administered in 2014–2015, and changes in technology since that time should be taken into account. Survey respondents estimated the frequency of their education and information use; however, self-reported data are vulnerable to recall bias and social desirability bias [60]. Individuals who complete surveys may have a more positive attitude toward research; therefore, our findings may overrepresent rural and remote nurses who use education and information, and overestimate the use of such sources. Also, while this analysis assessed the frequency with which certain sources of information were used, the nature and quality of the information applied in practice was not evaluated.

## CONCLUSIONS

Opportunities for rural and remote nurses to participate in and apply knowledge from continuing education and ongoing information use are important for safe and effective patient care. However, it is equally important that nurses critically reflect on new knowledge and, as much as possible, apply knowledge that is research-based. Future investigations could yield further insights into the specific forms of online/electronic information and education topics that are available to rural and remote nurses, and should explore how nurses in these communities evaluate and select the information that they ultimately use in practice. Moreover, further research about the factors associated with variations in education and information use according to registration status and practice setting would be valuable.

## FINANCIAL SUPPORT

The Nursing Practice in Rural and Remote Canada II study was funded by the Canadian Institutes of Health Research (MOP 130260).

## SUPPLEMENTAL FILE

Appendix Survey information and education sources questions

## 

**Julie Kosteniuk, PhD**, julie.kosteniuk@usask.ca, Professional Research Associate, Canadian Centre for Health and Safety in Agriculture, College of Medicine, University of Saskatchewan, Saskatoon, SK, Canada

**Norma J. Stewart, PhD, RN**, norma.stewart@usask.ca, Professor Emerita, College of Nursing, University of Saskatchewan, Saskatoon, SK, Canada

**Erin C. Wilson, PhD, NP(F)**, erin.wilson@unbc.ca, Assistant Professor, School of Nursing, University of Northern British Columbia, Prince George, BC, Canada

**Kelly L. Penz, PhD, RN**, kelly.penz@usask.ca, Assistant Professor, College of Nursing, University of Saskatchewan, Regina Campus, Regina, SK, Canada

**Ruth Martin-Misener, PhD, NP**, ruth.martin-misener@dal.ca, Professor, School of Nursing, Dalhousie University, Halifax, NS, Canada

**Debra G. Morgan, PhD, RN**, debra.morgan@usask.ca, Professor, Canadian Centre for Health and Safety in Agriculture, College of Medicine, University of Saskatchewan, Saskatoon, SK, Canada

**Chandima Karunanayake, PhD**, chandima.karunanayake@usask.ca, Professional Research Associate, Canadian Centre for Health and Safety in Agriculture, College of Medicine, University of Saskatchewan, Saskatoon, SK, Canada

**Martha L. P. MacLeod, PhD, RN**, macleod@unbc.ca, Professor and Northern Health – UNBC Knowledge Mobilization Research Chair, School of Nursing, University of Northern British Columbia, Prince George, BC, Canada
