# Supplementary material for: Communication tools and sources of education and information: a national survey of rural and remote nurses
Source: J Med Libr Assoc. 2019 Oct 1;107(4):538–54. doi: 10.5195/jmla.2019.632 (PMC6774559; doi:10.5195/jmla.2019.632)
Supplement: Appendix [file jmla-107-538-s001.pdf]

Journal of the Medical Library Association

Supplemental content to
J Med Libr Assoc. Oct;107(4):dx.doi.org/10.5195/jmla.2019.632
www.jmla.mlanet.org
© Kosteniuk, Stewart, Wilson, Penz, Martin-Misener, Morgan, Karunanayake, MacLeod 2019

Communication tools and sources of education and information: a national survey of rural and remote nurses

Julie Kosteniuk, PhD; Norma J. Stewart, PhD, RN: Erin C. Wilson, PhD, NP(F); Kelly L. Penz, PhD, RN; Ruth Martin-Misener, PhD, NP; Debra G. Morgan, PhD, RN; Chandima Karunanayake, PhD; Martha L. P. MacLeod, PhD, RN

APPENDIX

Survey information and education sources questions

G. INFORMATION AND EDUCATION SOURCES

G1) Do you have direct access in your primary workplace to the following information sources for your use in your nursing practice? *Mark all that apply.*

Yes = 1
No = 0
Missing = 96

G1A ○ High speed internet
G1B ○ Videoconference
G1C ○ Teleconference
G1D ○ Web conferencing (e.g., Skype, WebEx)
G1E ○ Electronic communication between health care providers (e.g., email, text)

G2) Please indicate how often you use education sources to update your nursing knowledge. These sources may include in-service training/ workplace education, continuing education, journal club, or nursing associations and colleges: *Missing = 96*

	Daily 6	At least once a week 5	At least once a month 4	At least once a year 3	Less than once a year 2	Never 1
a. In-person education sources (e.g., face-to-face)...... *G2A*	○	○	○	○	○	○
b. Online/Electronic sources (e.g., web-based, etc.)... *G2B*	○	○	○	○	○	○

G3) Please indicate how often you use the following information sources *to make specific decisions* in your nursing practice: *Missing = 96*

Online/electronic information sources (e.g., web-based, email, phone, text messaging, etc.)

	Daily 6	At least once a week 5	At least once a month 4	At least once a year 3	Less than once a year 2	Never 1
a. Policies, protocols, standards, or regulatory tools.......... *G3A*	○	○	○	○	○	○
b. Clinical practice guidelines *G3B*	○	○	○	○	○	○
c. Nursing/medical journals *G3C*	○	○	○	○	○	○
d. Nursing/medical textbooks *G3D*	○	○	○	○	○	○
e. Internet search engines (e.g., Google, Yahoo) *G3E*	○	○	○	○	○	○
f. Practice support resources (e.g., NurseOne, UpToDate, eMedicine)..... *G3F*	○	○	○	○	○	○
g. Research databases (e.g., CINAHL, Medline, PubMed)....... *G3F*	○	○	○	○	○	○

G4) Please indicate how often you use the following information sources *to make specific decisions* in your nursing practice: *Missing = 96*

Print/paper format information sources

	Daily 6	At least once a week 5	At least once a month 4	At least once a year 3	Less than once a year 2	Never 1
a. Policies, protocols, standards, or regulatory tools.......... *G4A*	○	○	○	○	○	○
b. Clinical practice guidelines *G4B*	○	○	○	○	○	○
c. Nursing/medical journals *G4C*	○	○	○	○	○	○
d. Nursing/medical textbooks *G4D*	○	○	○	○	○	○

16
